# The Role of Chitosan Oligosaccharide in Metabolic Syndrome: A Review of Possible Mechanisms

**DOI:** 10.3390/md19090501

**Published:** 2021-09-01

**Authors:** Wenjing Tao, Geng Wang, Jintao Wei

**Affiliations:** 1 Hubei Key Laboratory of Animal Embryo and Molecular Breeding, Institute of Animal Husbandry and Veterinary, Hubei Academy of Agricultural Sciences, Wuhan 430064, China; taowenjing1127@163.com; 2 Key Laboratory of Molecular Animal Nutrition, Ministry of Education, College of Animal Science, Zhejiang University, Hangzhou 310058, China; wanggeng@zju.edu.cn

**Keywords:** chitosan oligosaccharide, metabolic syndrome, obesity, diabetes, hypertension

## Abstract

Metabolic syndrome, a cluster of metabolic disorders including central obesity, insulin resistance, hyperglycemia, dyslipidemia, and hypertension, has become a major public health problem worldwide. It is of great significance to develop natural products to prevent and treat metabolic syndrome. Chitosan oligosaccharide (COS) is an oligomer of chitosan prepared by the deacetylation of chitin, which is the second most abundant polymer in nature. In recent years, COS has received widespread attention due to its various biological activities. The present review will summarize the evidence from both in vitro and in vivo studies of the beneficial effects of COS on obesity, dyslipidemia, diabetes mellitus, hyperglycemia, and hypertension, and focus attention on possible mechanisms of the prevention and treatment of metabolic syndrome by COS.

## 1. Introduction

Metabolic syndrome, which is a cluster of metabolic disorders including central obesity, insulin resistance, hyperglycemia, dyslipidemia, and hypertension, has become a major public health problem worldwide, and its global prevalence is estimated to be 25% [1,2]. Different clinical criteria for metabolic syndrome have been developed over time by various organizations, such as the World Health Organization (WTO), the National Cholesterol Education Program Adult Treatment Panel III (NCEP–ATP III), and the International Diabetes Federation (IDF) [3]. In 2009, a consensus statement was published by the IDF and the American Heart Association/National Heart, Lung, and Blood Institute (AHA/NHLBI) that metabolic syndrome is diagnosed by the co-occurrence of three or more of the following criteria: (1) a waist circumference over 101.6 cm for men or 88.9 cm for women; (2) fasting blood sugar over 100 mg/dL; (3) a fasting triglycerides (TG) level over 150 mg/dL; (4) a fasting high-density lipoprotein-cholesterol (HDL-C) level less than 40 mg/dL for men or 50 mg/dL for women; (5) blood pressure over 130/85 mmHg [4]. Metabolic syndrome has been increasingly recognized as a risk factor for cardiovascular disease and type 2 diabetes [5,6]. In addition, metabolic syndrome is found to be associated with non-alcoholic fatty liver, chronic kidney disease, osteoporosis, and even cancer [7,8,9,10]. Currently, the main interventions for metabolic syndrome consist of lifestyle alteration and pharmacologic therapies [11,12]. In many cases, lifestyle alteration alone may not achieve the best outcomes, and therefore drugs are often used. However, these drugs have many side effects, such as gastrointestinal upset, hypoglycemia, and myalgia [13,14]. In addition, their applications are limited due to their high cost. Therefore, searching for natural products to reduce the risk and progression of metabolic syndrome has attracted increasing attention. 

Chitosan oligosaccharide (COS) is the degradation product of chitosan, prepared by the deacetylation of chitin, which is the second most abundant polymer in nature found in the shells of crustaceans and shells, as well as in the cell walls of fungi [15,16]. COS has been widely applied in multiple fields, including biomedicine, food, cosmetics, agriculture, and animal husbandry [17]. Numerous studies have reported that COS has anti-inflammatory, anti-oxidative, antimicrobial, immunostimulatory, anti-obesity, hypolipidemic, hypoglycemic, and anti-hypertensive activities [18,19]. Therefore, COS may be a promising natural product for the prevention or treatment of metabolic syndrome. 

In this review, we will summarize the evidence from both in vitro and in vivo studies of the beneficial effects of COS on obesity, dyslipidemia, diabetes mellitus, hyperglycemia, and hypertension, and address possible mechanisms of the prevention and treatment of metabolic syndrome by COS.

## 2. Characterization of COS

COS, composed of β-(1,4)-linked D-glucosamine, is a mixture of oligomers of chitosan with a degree of polymerization (DP) ≤ 55 and an average molecular weight (MW) ≤ 10 kDa [19]. COS is usually prepared by hydrolysis of chitosan, which is a deacetylated product of chitin [20]. Specifically, chitin is treated with 10–15 M NaOH at 40–100 °C for several hours to several days to prepare chitosan, and then COS can be obtained from chitosan via hydrolysis reactions, which can be catalyzed by physical, chemical, or enzymatic methods [21]. Among them, the enzymatic method has been extensively studied and well developed because it is comparatively more controllable and effective [21]. The specific enzymes, including chitosanases and chitinases, are very effective, but their applications are limited due to their high cost. Commercially available COS is usually prepared using nonspecific enzymes, including pectinases, cellulases, deacetylases, amylases, lipases, and proteases [22].

Due to the lack of digestive enzymes, which can hydrolyze β-1,4-glycosidic bonds, in the gastrointestinal tract of animals and humans, COS cannot be degraded in the gastrointestinal tract. Accumulated evidence indicates that COS is well absorbed via the intestinal epithelia. An in vitro study revealed that COS could be transported through a monolayer of Caco-2 cells, and as the MW of COS decreased, the absorption rate increased [23]. In rats, the plasma concentration of COS reached its peak value 30 min after oral administration, and the intestinal absorption rate of COS was also negatively correlated with its MW [23]. COS can be absorbed from the small intestine into the blood mainly by a combination of carrier protein and free diffusion, and after being absorbed, COS is mainly distributed to the liver, kidney, and spleen [24]. The absorbed COS can be degraded to a lower MW by lysozyme in the blood, liver, kidney, and urine, whereas the unabsorbed COS can reach the distal intestine to be fermented and utilized by gut microbiota [24]. The kidney plays a key role in the excretion of COS, and COS is mainly excreted with urine [19].

## 3. Effect and Mechanism of COS on Metabolic Syndrome

### 3.1. Effect and Mechanism of COS on Obesity and Dyslipidemia

**Figure 1:**
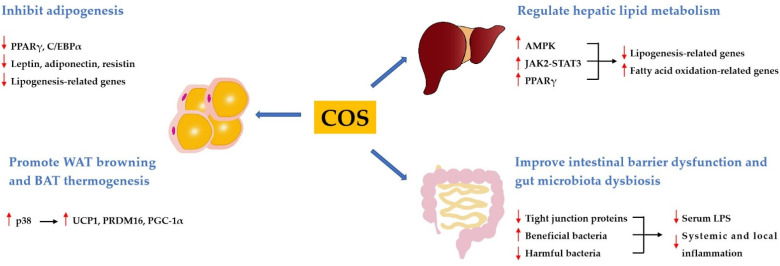
Mechanisms involved in the regulation of obesity and dyslipidemia by COS.

Obesity is a chronically trophic metabolic disease characterized by excessive body fat accumulation resulting from energy imbalance. Obesity is usually associated with dyslipidemia, including hypertriglyceridemia, and hypercholesterolemia. Various studies have shown that COS had good anti-obese and hypolipidemic activities. It has been demonstrated that COS was effective in reducing body weight gain, lowering blood lipid levels, decreasing hepatic fat accumulation, and inhibiting adipocyte hyperplasia and hypertrophy in high-fat diet (HFD)-induced obese rats or mice [25,26,27,28,29,30]. In addition, COS has been found to decrease body weight gain and plasma lipids in ob/ob mice [31]. These findings imply that COS may potentially be used to prevent or treat obesity and dyslipidemia. The mechanisms underlying the anti-obese and hypolipidemic activities of COS include the inhibition of adipogenesis, the promotion of white adipose tissue (WAT) browning and brown adipose tissue (BAT) thermogenesis, the regulation of hepatic lipid metabolism, the improvement of the intestinal barrier dysfunction, and gut microbiota dysbiosis (Figure 1).

#### 3.1.1. COS Inhibits Adipogenesis

Obesity is accompanied by excessive lipid storage in adipose tissue, which is determined primarily by adipocyte hyperplasia or hypertrophy [32]. Adipogenesis, as the basis of hyperplasia, is the transformation process of preadipocytes into mature adipocytes [33]. The inhibition of adipogenesis is a potential strategy for the prevention and treatment of obesity. In vitro studies showed that COS could inhibit the differentiation of 3T3-L1 preadipocytes and decrease lipid accumulation by downregulating the expressions of peroxisome proliferator-activated receptor γ (PPARγ) and CCAAT enhancer-binding proteins α (C/EBPα), two key adipogenesis-related transcription factors [34,35]. Moreover, COS downregulated the expressions of related molecules in 3T3-L1 adipocyte, such as leptin, adiponectin, resistin, fatty acid binding protein (FABP), fatty acid synthase (FAS), and glucose transporter 4 (GLUT4) [30,34,36]. Interestingly, Bahar et al. reported that COS inhibited the de-methylation of leptin gene promoter in 3T3-L1 adipocytes, indicating that COS might suppress adipocyte differentiation via epigenetic mechanisms [37]. Furthermore, COS inhibited adipocyte hyperplasia and hypertrophy in HFD-fed mice or rats by regulating the expression of lipogenesis-related genes in the adipose tissue, and the mechanisms underlying the anti-adipogenic effect of COS involved the inhibition of the PPAR-γ signaling pathway [25,26,27].

#### 3.1.2. COS Promotes WAT Browning and BAT Thermogenesis

Adipose tissues play important roles in the regulation of energy homeostasis. WAT functions to store energy and release adipokines in response to various stimuli, whereas BAT serves as a thermogenic tissue to regulate body temperature [38]. WAT is able to switch to a brown phenotype, and this process is called browning [39]. Therefore, increasing WAT browning and BAT thermogenesis are expected to be promising methods to prevent obesity. Wang et al. found that COS reduced weight gain and serum lipid levels, and increased energy expenditure and brown fat content in HFD-induced obese rats [40]. Additionally, this study revealed that COS promoted WAT browning and BAT thermogenesis by upregulating the expressions of uncoupling protein 1 (UCP1), PRD1-BF1-RIZ1 homologous domain-containing 16 (PRDM16), and peroxisome proliferator-activated receptor-γ coactivator-1α (PGC-1α) in these two types of adipose tissues through activating the p38 signaling pathway.

#### 3.1.3. COS Regulates Hepatic Lipid Metabolism 

The liver is the central organ of lipid metabolism. An in vitro study has shown that COS ameliorated lipid accumulation in palmitic acid-induced HepG2 cells via activating the PPARγ signaling pathway [28]. It has been demonstrated that COS was effective in inhibiting serum activities of alanine aminotransferase and aspartate aminotransferase, reducing hepatic lipid accumulation, and decreasing hepatic steatosis in HFD-induced obese rats or mice [26,28,41,42]. Additionally, these studies revealed that COS alleviated hepatic lipid metabolism disorder by downregulating the expressions of lipogenesis-related genes, including sterol regulatory element-binding protein-1c (SREBP-1c), FAS, acetyl-CoA carboxylase (ACC), and 3-hydroxy-3-methylglutaryl-CoA reductase (HMGCR), and upregulating the expressions of fatty acid oxidation-related genes, including PPARα and carnitine palmitoyl transferase 1 (CPT-1). Further studies revealed that these beneficial effects were mediated by the activation of the adenosine monophosphate-activated protein kinase (AMPK), Janus kinase-2-signal transducer, and activators of transcription-3 (JAK2-STAT3) and PPARγ signaling pathways.

#### 3.1.4. COS Improves Intestinal Barrier Dysfunction and Gut Microbiota Dysbiosis

It is well known that gut microbiota can affect host physiology, and gut microbiota disorder along with disturbance of intestinal barrier integrity are involved in the development of obesity and dyslipidemia [43]. He et al. demonstrated that COS reduced body weight gain and serum lipid levels, and improved intestinal barrier dysfunction and gut microbiota dysbiosis in HFD-fed mice [29]. Specifically, at phylum level, COS increased the ratio of Firmicutes to Bacteroidetes, and the abundance of Proteobacteria and Actinobacteria. At genus level, COS decreased the relative abundance of inflammatory bacteria such as *Erysipelatoclostridium* and *Alistipes*, and increased the abundance of beneficial intestinal bacteria such as *Akkermansia* and *Gammaproteobacteria*. Spearman’s correlation analysis demonstrated that the anti-obese and hypolipidemic activities of COS were related to the modification of gut microbiota. Notably, intestinal barrier dysfunction and gut microbiota dysbiosis were involved in the leakage of lipopolysaccharide (LPS), a component of the cell wall of Gram-negative bacteria, into the blood to cause inflammation [44]. Chronic and low-grade inflammation is a common feature of obesity [45]. Various studies have demonstrated that COS suppressed the serum LPS level, as well as the inflammation in the blood, liver, adipose tissue, and colon of HFD-induced obese mice or rats [27,28,29]. These effects were ascribed to the improvement of intestinal barrier dysfunction and gut microbiota dysbiosis. In general, COS may prevent obesity, dyslipidemia, and accompanied inflammation via improving intestinal barrier dysfunction and normalizing the dysbiosis of gut microbiota.

### 3.2. Effect and Mechanism of COS on Diabetes Mellitus and Hyperglycemia

**Figure 2:**
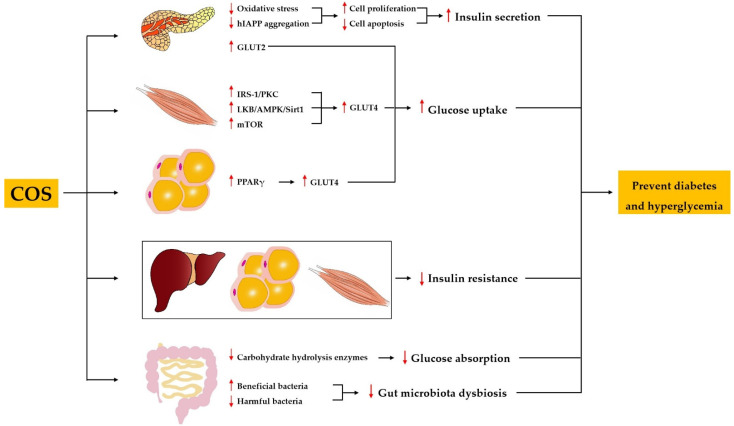
Mechanisms involved in the regulation of diabetes and hyperglycemia by COS.

Diabetes mellitus is characterized by chronic hyperglycemia resulting from inadequate secretion or inefficient utilization of insulin [46]. The anti-diabetic activity of COS has been demonstrated using various types of diabetic models. The administration of COS improved the general situation and diabetic symptoms, decreased the levels of blood glucose and urine glucose, and normalized impaired glucose tolerance in neonatal streptozotocin (STZ)-induced type 2 diabetic rats, a model of non-insulin-dependent diabetes mellitus [47,48]. Ju et al. showed that COS treatment for 8 weeks resulted in decreased fasting blood glucose and fasting insulin levels as well as an increased insulin sensitivity index and improved oral glucose tolerance in insulin-resistant rats induced by a high-energy diet together with STZ [49]. Katiyar et al. reported that COS was effective in decreasing blood glucose and improving renal dysfunction in alloxan-induced diabetic mice [50]. In addition, COS has been found to decrease the blood glucose in db/db mice [51,52]. A randomized, double-blind, placebo-controlled clinical trial was conducted by Kim et al. to evaluate the effect of COS on glucose control in subjects with prediabetes. The results showed that COS supplementation of 1500 mg per day for 12 weeks in Korean subjects between the ages of 20 and 75 years significantly decreased the postprandial serum glucose level [53]. These findings imply that COS could be used as a natural agent for the prevention and treatment of diabetes mellitus and hyperglycemia. The mechanisms underlying the anti-diabetic and anti-hyperglycemic activity of COS include the protection of pancreatic β cells and the promotion of insulin secretion, the alleviation of insulin resistance, the inhibition of carbohydrate-hydrolyzing enzymes, the promotion of glucose uptake, and the improvement of gut microbiota dysbiosis (Figure 2).

#### 3.2.1. COS Protects Pancreatic β Cells and Promotes Insulin Secretion

An adequate population of healthy pancreatic β cells is of high importance for sufficient insulin secretion. An in vivo study found that loss of pancreatic cells, nuclear pyknosis of pancreatic cells, and atrophy of pancreatic islets were minimized by COS in STZ-induced diabetic rats [54]. Similarly, COS recovered the atrophied islet and increased the pancreas-to-body weight ratio in diabetic rats induced by a high-energy diet together with STZ [49]. Moreover, in vitro studies have shown that COS can promote cell proliferation and ameliorate STZ-induced cell apoptosis in a pancreatic β cell line [48,49]. Increased oxidative stress is one of the main causes of loss and dysfunction of pancreatic β cells. Yuan et al. reported that COS improved the activities of total antioxidant capacity (T-AOC) and superoxide dismutase (SOD), and decreased the content of malondialdehyde (MDA) in serum of STZ-induced rats [54]. Similarly, Ju et al. found that COS increased the SOD activity and reduced the MDA content in pancreas homogenate of STZ-induced rats [49]. In addition, Karadeniz et al. demonstrated that COS could exhibit free radical scavenging activity to protect against hydrogen peroxide (H_2_O_2_)-induced oxidative stress in β cells [55]. These findings suggest that COS may protect pancreatic β cells by improving the activities of antioxidative enzymes or acting as free radical scavengers. Interestingly, a recent study reported that COS could inhibit the aggregation of human islet amyloid polypeptide (hIAPP) and destroy the formed hIAPP fibrils, thus alleviating hIAPP-induced cytotoxicity, apoptosis, and cell cycle arrest of mouse β cells [56]. hIAPP is secreted by β cells in the pancreas and plays an important role in regulating glucose metabolism [57]. The abnormal aggregation of hIAPP is considered to be responsible for the loss and dysfunction of pancreatic β cells in patients with type 2 diabetes [58]. It can be concluded that as an antidiabetic agent, COS may protect pancreatic β cells by suppressing oxidative stress and inhibiting hIAPP aggregation.

Further studies showed that COS not only protected pancreatic β cells, but more importantly promoted insulin secretion. It has been reported that COS promoted insulin secretion in primary cultured rat pancreatic cells [48]. Ju et al. found that COS enhanced glucose stimulated insulin secretion in rat pancreatic β cell lines [49]. In vivo studies demonstrated that COS could increase insulin secretion in STZ-induced diabetic rats, as well as db/db mice [49,52].

#### 3.2.2. COS Alleviates Insulin Resistance

Insulin resistance is one of the earliest manifestations of diabetes, and it refers to reduced sensitivity and response to insulin in target tissues, including the liver, skeletal muscle, and adipose tissue [59]. Ju et al. found that the insulin sensitivity index and glucose tolerance in high-energy diet together with STZ-induced diabetic rats were improved by COS supplementation [49]. A study with db/db mice also indicated that COS significantly reversed insulin resistance [52]. Many key components in the insulin signaling pathway have been identified, such as the insulin receptor, insulin receptor substrate (IRS), phosphoinositide 3-kinase (PI3K), and Akt [60]. However, the molecular mechanisms of COS alleviating insulin resistance are still lacking, and further research is needed.

#### 3.2.3. COS Inhibits Carbohydrate-Hydrolyzing Enzymes

Carbohydrate-hydrolyzing enzymes can break down carbohydrate into monosaccharides, and inhibiting the activities of intestinal carbohydrate-hydrolyzing enzymes can result in a reduction of glucose absorption in the small intestine, thus decreasing the blood glucose level. Therefore, the inhibitors of carbohydrate-hydrolyzing enzymes are potential candidates for the treatment of diabetes. Long-term supplementation of COS significantly reduced the levels of blood glucose and glycated hemoglobin A1c (HbA1c) by inhibiting the activities of intestinal sucrase and glucoamylase, and downregulating the mRNA expression of the sucrase–isomaltase (SI) complex in db/db mice [51]. Corresponding with these findings, an in vitro study demonstrated that the activities of rat intestinal α-glucosidase and porcine pancreatic α-amylase were inhibited by COS with a different MW [61]. In addition, COS with a MW < 1 kDa had a stronger ability to control postprandial blood glucose levels in Sprague–Dawley (SD) rats, as it was more readily absorbed into the bloodstream than COS with a higher MW. It is reported that COS inhibited the activities of α-glucosidase and downregulated the mRNA expression of the SI complex in human intestinal cells [62]. All these observations might indicate that COS could be used for the prevention of diabetes via inhibition of intestinal carbohydrate hydrolysis enzymes. 

#### 3.2.4. COS Promotes Glucose Uptake

GLUT4 is the main glucose transporter in skeletal muscle and adipose tissue, whereas GLUT2 is the main glucose transporter in pancreatic islets [63]. It has been reported that COS upregulated the expression of GLUT4 in muscle and adipose tissue of diabetic rats, and an in vitro study demonstrated that COS also upregulated the expression of GLUT2 in pancreatic β cell lines [49]. Moreover, COS enhanced glucose uptake in C2C12 myotubes by upregulating GLUT4 expression through the activation of the IRS-1/PKC, LKB/AMPK/Sirt1, as well as mTOR pathways [64]. Yu et al. found that COS increased glucose uptake in adipocytes by upregulating GLUT4 expression via activation of the PPARγ signaling pathway [62]. 

#### 3.2.5. COS Improves Gut Microbiota Dysbiosis

The dysbiosis of gut microbiota is strongly associated with the onset and development of diabetes [65,66]. Therefore, the modulation of gut microbiota may be a novel strategy for prevention and treatment of diabetes and its comorbidities. Zheng et al. demonstrated that COS decreased the blood glucose level, inhibited destruction of gut integrity, and reversed the dysbiosis of gut microbiota in diabetic db/db mice [52]. At phylum level, COS treatment dramatically suppressed the growth of Firmicutes and increased the population of Bacteroidetes in db/db mice. Furthermore, at genus level, COS treatment remarkably reduced the abundance of *Lachnospiraceae NK4A136 group*, *Alistipes*, *Helicobacter*, *Ruminococcus_1*, and *Odoribacter*, and increased the abundance of *Lachnospiraceae_UCG_001* and *Akkermansia*. Zheng et al. utilized Spearman’s correlation analysis to demonstrate the association of COS-modulated bacteria and metabolic biomarkers. The Phylogenetic Investigation of Communities by Reconstruction of Unobserved States (PICRUSt) analysis indicated that COS treatment may have restrained motility capacity, reduced oxidative stress, and regulated the metabolic pathways of gut microbiota in diabetic db/db mice. Similarly, COS reduced hyperglycemia by regulating the gut microbiota in STZ-induced diabetic mice [67]. Specifically, COS increased the relative abundance of Firmicutes to Bacteroidetes. At the order level, COS increased the abundance of beneficial bacteria *Verrucomicrobiales*, and decreased the abundance of harmful bacteria *Proteobacteria*. In general, COS may reverse the abnormal metabolic profiles of diabetes via normalizing the dysbiosis of gut microbiota.

### 3.3. Effect and Mechanism of COS on Hypertension

Hypertension is considered a leading risk factor for the development of cardiovascular disease, chronic kidney disease, and cognitive impairment [68,69,70]. The pathophysiology of hypertension is associated with several factors, including genetics, the activation of the renin–angiotensin–aldosterone system (RAAS), the activation of the sympathetic nervous system, insulin resistance, vascular remodeling, endothelial dysfunction, and impaired ion channels [71]. Hong et al. reported that a single oral dose of chitosan trimer (2.14 mg/kg) significantly reduced blood pressure in spontaneously hypertensive rat models [72]. The anti-hypertensive activity of COS might be related to the blocking of RAAS and the improvement of endothelial dysfunction.

#### 3.3.1. COS Blocks RAAS

The RAAS plays an important role in the regulation of blood pressure, and blockers of RAAS are effective approaches for the treatment of hypertension [73]. Renin, synthesized, stored, and released from the renal juxtaglomerular cells, is a rate-limiting enzyme in RAAS, and its main function is to catalyze the hydrolysis of angiotensinogen to form angiotensin I [74]. It was found that renin activities were inhibited by COS with a different MW and degree of deacetylation (DD) [75]. Additionally, this study revealed that COS with an average MW of 1–5 kDa had higher renin-inhibitory activity than COS with an average MW of <1 kDa or 5–10 kDa, and COS with a DD of 90% had higher renin-inhibitory activity than COS with a DD of 50%.

The angiotensin-I converting enzyme (ACE), as a key component of RAAS, can transform angiotensin I into vasoconstrictor angiotensin II, and degrade vasodilator bradykinin [76]. Therefore, ACE inhibitors have been developed to control blood pressure. Hong et al. found that ACE activities were inhibited by COS with varying DP from 1 to 10, and among all oligosaccharides, the chitosan trimer (DP = 3) exhibited the highest inhibitory activity [72]. Furthermore, an in vivo study demonstrated that chitosan trimer reduced blood pressure in hypertensive rats. The ACE inhibitory activity of COS also depends on its MW and DD. Park et al. demonstrated that COS with an average MW of 1–5 kDa has higher ACE inhibitory activity than COS with an average MW of <1 kDa or 5–10 kDa [77]. Notably, this study revealed that the ACE inhibitory activity of COS was increased as the DD decreased from 90% to 50%. In addition, it was found that several types of chemical modifications of COS, including carboxylated COS, sulfated COS, and aminoethyl-conjugated COS, exhibited higher ACE inhibitory activity than unmodified COS, as increased negative charge density of modified COS contributed to enhanced binding of COS to the ACE obligatory active site [78,79,80].

#### 3.3.2. COS Improves Endothelial Dysfunction

Endothelial dysfunction plays a pivotal role in the development of many cardiovascular diseases, including hypertension [81]. Increased oxidative stress is considered one of the leading causes of endothelial dysfunction. Liu et al. reported that COS attenuated H_2_O_2_-induced oxidative stress in endothelial cells via decreasing the intracellular reactive oxygen species (ROS), suppressing the production of MDA, restoring the activities of endogenous antioxidants, increasing the levels of nitric oxide (NO) and nitric oxide synthase (NOS), and reducing cell apoptosis [82]. On the other hand, inflammation is known to be associated with endothelial dysfunction, and can contribute to hypertension. COS inhibited LPS-induced inflammation in endothelial cells by downregulating the expression of inflammatory cytokines including interleukin-6 (IL-6) and IL-8, and adhesion molecules including E-selectin and intercellular adhesion molecule-1 (ICAM-1) through inhibiting the p38, ERK1/2, PI3K/Akt, and nuclear transcription factor κB (NF-κB) signaling pathways [83,84,85]. Further, evidence from both in vivo and in vitro studies conducted by Li et al. has shown that COS attenuated LPS-induced vascular endothelial inflammatory response through decreasing O-GlcNAc transferase-dependent O-GlcNAcylation of NF-κB [86]. These findings showed that COS could improve endothelial dysfunction by suppressing inflammation and oxidative stress, thereby exhibiting anti-hypertensive activity.

## 4. Conclusions

Metabolic syndrome has become a major public health problem worldwide. In recent years, natural products have attracted increasing attention to reduce the risk and progression of metabolic syndrome. According to the various studies analyzed in this review, COS has beneficial effects on various components of metabolic syndrome, including obesity, dyslipidemia, diabetes mellitus, hyperglycemia, and hypertension. COS prevents obesity and dyslipidemia mainly by inhibiting adipogenesis, promoting WAT browning and BAT thermogenesis, regulating hepatic lipid metabolism, and improving intestinal barrier dysfunction and gut microbiota dysbiosis. Additionally, it prevents diabetes mellitus and hyperglycemia by protecting pancreatic β cells and promoting insulin secretion, alleviating insulin resistance, inhibiting carbohydrate-hydrolyzing enzymes, promoting glucose uptake, and improving gut microbiota dysbiosis. Furthermore, it prevents hypertension by blocking RAAS and improving endothelial dysfunction. Collectively, COS could protect major tissues, including the pancreas, muscle, adipose tissue, liver, intestine, and blood vessels against injury, and function in a multi-targeted manner to mitigate metabolic syndrome. Nevertheless, more studies are still needed to further reveal the molecular mechanism and action targets of COS. 

Although the majority of studies have reported the beneficial effects of COS on metabolic syndrome, there are also some studies that show its adverse effects. Chiu et al. reported that diets supplemented with 5% COS for eight weeks may induce liver injury in HFD-induced obese rats [87]. Moreover, Eisa et al. demonstrated the toxicity and teratogenic effects of COS on female rats and their progenies [88]. An in vitro study showed that COS with a concentration above 70 μg/mL induced strong cytotoxic effect in human lymphocytes [89]. Therefore, more studies on the safety assessments of COS should be performed, and it is essential to determine the optimal dosage of COS as a dietary supplement. In addition, most works hitherto were carried out in the cell or rodent models, therefore further clinical trials will be important for the application of COS to reduce the risk and progression of metabolic syndrome.
